# Evaluation of Antioxidant Capacity and Synergistic Associations of Quinonemethide Triterpenes and Phenolic Substances from *Maytenus ilicifolia* (Celastraceae)

**DOI:** 10.3390/molecules15106956

**Published:** 2010-10-11

**Authors:** Vânia Aparecida de Freitas Formenton Macedo dos Santos, Daniela Pereira dos Santos, Ian Castro-Gamboa, Maria Valnice Boldrin Zanoni, Maysa Furlan

**Affiliations:** 1 Núcleo de Bioensaios, Biossíntese e Ecofisiologia de Produtos Naturais – NuBBE – Departamento de Química Orgânica, Instituto de Química, Universidade Estadual Paulista, UNESP, CP 355,14801-970 Araraquara-SP, Brazil; 2 Departamento de Química Analítica, Instituto de Química, Universidade Estadual Paulista, UNESP, CP 355, 14801-970 Araraquara-SP, Brazil

**Keywords:** antioxidant, high-performance liquid chromatography coupled with electrochemical detection (HPLC-EICD), cyclic voltammetry (CV), quinonemethide, flavonoid

## Abstract

This work describes the isolation of the secondary metabolites identified as the quinonemethides maytenin (**1**) and pristimerin (**2**) from *Maytenus ilicifolia* extracts obtained from root barks of adult plants and roots of seedlings and their quantification by high performance liquid chromatography coupled to a diode array detector. The electrochemical profiles obtained from cyclic voltammetry and a coulometric detector coupled to high-performance liquid chromatography contributed to the evaluation of their antioxidant capacity. The antioxidant properties of individual components and the crude extracts of the root barks of *Maytenus ilicifolia* were compared and the possible synergistic associations of quinonemethide triterpenes and phenolic substances were investigated by using rutin as a model phenolic compound.

## 1. Introduction

*Maytenus ilicifolia* (Celastraceae) is a native Tropical Atlantic Forest plant widely used in traditional medicine as an anti-inflammatory, analgesic and antiulcerogenic [1,2,3]. Its pharmacological properties have being reviewed in literature [4,5,6,7]. Previous investigations have shown the occurrence in the species of several secondary metabolites [3,7], including quinonemethide triterpenes [8,9,10] and sesquiterpene pyridine alkaloids [11,12] with a wide spectrum of biological activities. Studies of the antioxidant activity of flavonoids and quinonemethide triterpenes using HPLC methods [13,14,15] and their biological activities [16,17,18,19,20,21,22,23,24,25,26,27,28,29,30], have also been reported, but knowledge about byproducts other than those of plant origin is scarce. Taking into consideration that the knowledge about the variability of secondary metabolites present in extracts of plants is important, and such substances can act in synergy in the protection of cells and tissues, assays able to provide this information are in high demand. 

In addition to several other methods proposed to evaluate antioxidant properties [31,32,33,34,35,36,37,38,39,40], the use of electrochemical methods has been demonstrated to be a useful alternative to integrated antioxidant capacity [41,42,43,44] (cyclic voltammetry) and identification and quantification by high-performance liquid chromatography coupled with electrochemical detection (HPLC-ED) [45,46,47]. The electrochemical response is directly related to the structure of the antioxidant and the potential required for its oxidation [47]. This can be an excellent option for all antioxidants that usually present redox properties. The HPLC-ED technique can also offer the possibility to increase the sensibility and selectivity of antioxidant determination methods in complex matrices [48,49].

This work describes the synergistic associations of two secondary metabolites, identified as the quinonemethide triterpenes maytenin (**1**) and pristimerin (**2**), previously isolated from seedlings and adult plants of *Maytenus ilicifolia* root barks and their antioxidant properties as monitored by cyclic voltammetry and high performance liquid chromatography coupled to electrochemical detection.

## 2. Results and Discussion

### 2.1. HPLC-DAD analyses

**Figure 1:**
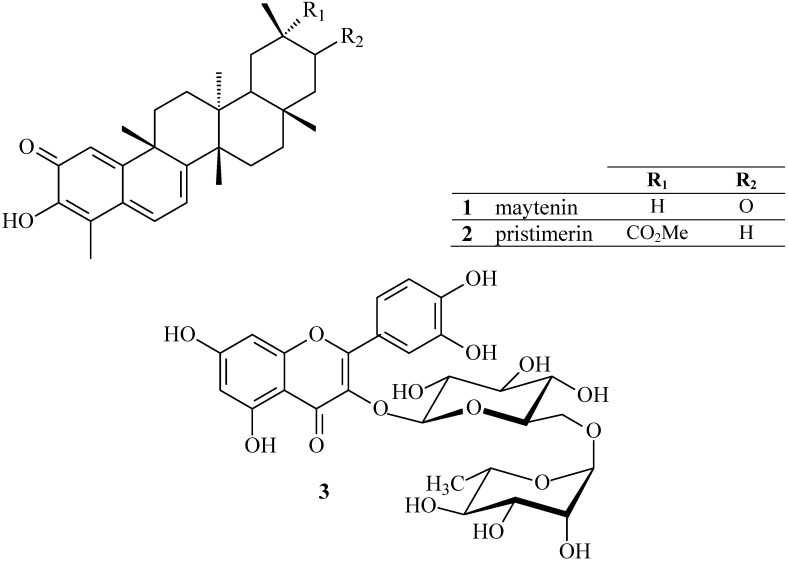
Structures of the isolated quinonemethide triterpenes of *Maytenus ilicifolia*: maytenin (**1**); pristimerin (**2**) and rutin (**3**).

Chromatographic fractionation of the extract obtained from root barks of *Maytenus ilicifolia* resulted in the isolation of compounds **1** and **2** (Figure 1, Curve A) [24,25].

**Figure 2:**
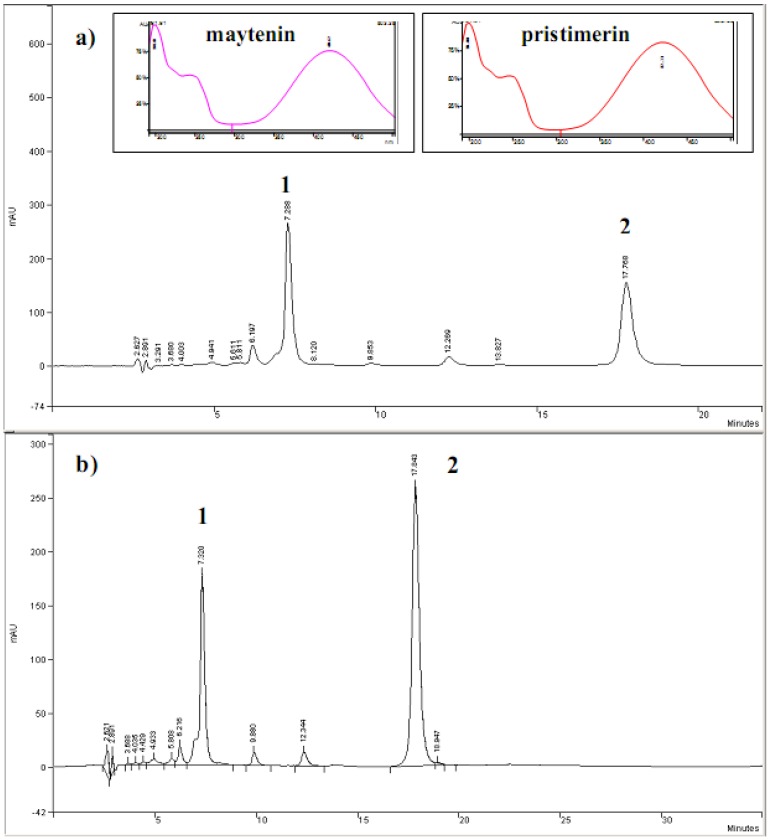
Chromatograms of HPLC-DAD for extracts obtained from root barks of the adult plants (a) and roots of seedlings (b) of *Maytenus ilicifolia*. Identification of peaks: **1**, maytenin; **2**, pristimerin. Insert spectra: UV-Vis spectra correspondent to peaks 1 and 2. λ= 420 nm.

Extracts from root barks of adult plant (E_2_) (Curve a) and root of seedlings (E_4_) (Curve b) of *Maytenus ilicifolia* were submitted to chromatographic analysis using HPLC-DAD based on compounds **1** and **2** as standard samples, as shown in Figure 2. The quinonemethide triterpenes **1** and **2** present retention times of 7.3 min and 17.8 min, respectively, and were characterized by their absorbance at 420 nm (insert of Figure 2) and ES-MS spectra [17]. 

The extracts from root barks of adult plant (E2) also presented a defined peak at a retention time of 18.9 min; with detection at 254 nm. The UV spectra recorded from HPLC peaks, corroborated the identification of flavonoids behaviour [51]. The class of the flavonoid can also be distinguished on the basis of the absorption in the 300–400 nm region (Band I, cinnamoyl system). The saturation at C-2 and C-3 (flavanones and flavanonols), as well as the lack of conjugation between rings A and B (isoflavones), leads to the disappearance of the maximum near 350 nm. In contrast, the presence of the double bond causes a maximum that is higher in flavones than in flavonols. Different aglycones within the same class can be distinguished by comparing their on-line UV spectra in the 250–290 nm region (Band II, benzoyl system), as the shape of the maximum is strictly related to the substitution pattern.

The analytical curves were constructed from HPLC-DAD data obtained for isolated compounds **1** and **2**, using optimized conditions. A linear relationship was obtained in all the concentration range, from 12.5 μg mL^-1^ to 100 μg mL^-1^, following the equation: Area = -117437 + 43347.18 C (C = μg mL^-1^), r = 0.999 for maytenin (**1**) and Area = -95866.6087 + 17446.2956 C (C = μg mL^-1^), r = 0.999 for pristimerin (**2**). From these calibration curves it was possible to determine the quantity of **1** and **2** compounds in the extracts of root barks of adult plant (E_2_). These amounts in the sample were 3.84% and 14.08% (m/m) for compounds **1** and **2**, respectively.

**Figure 3:**
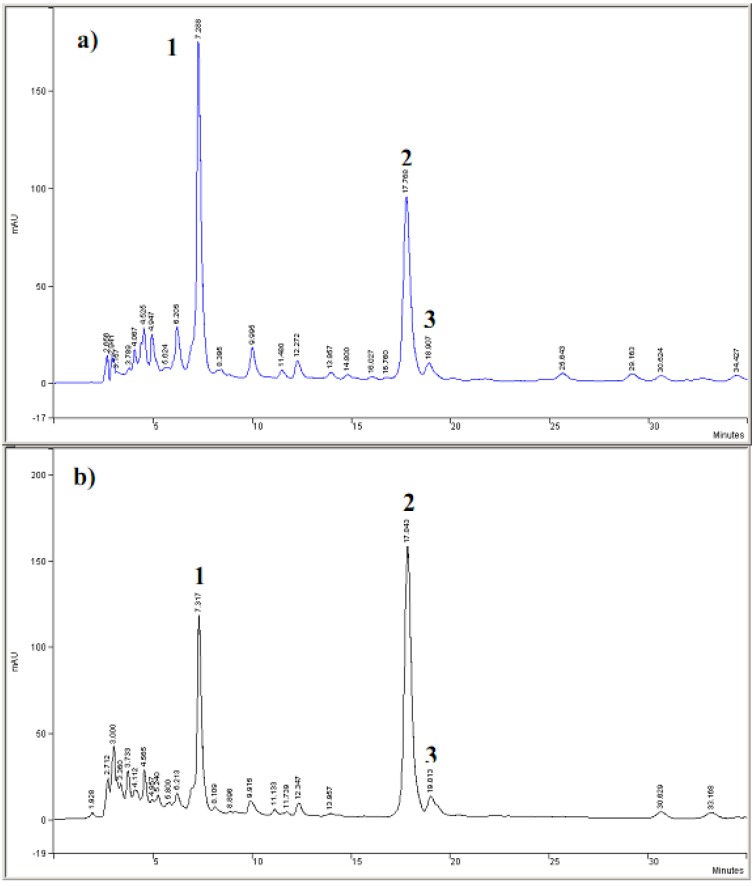
Chromatograms HPLC-DAD obtained for root of *Maytenus ilicifolia* extracts. Identification of peaks: 1, maytenin; 2, pristimerin. Peak 3 was identified by the comparison of UV spectra with data literature. a) extracts of root barks of the adults plants and b) root of seedling.

The HPLC-DAD obtained for extract of root of seedlings (E_4_) is shown in Figure 3. It is possible to detect the occurence of compounds **1** and **2**, at the retention times of 7.3 and 17.8 min. The occurrence of a peak at a retention time of 19.01 min. also indicated the presence of flavonoids with an absorption peak around 300–400 nm due the ring B of flavonoids (insaturation C-2 and C-3). The concentration of **1** and **2** accumulated in the root of seedlings extract (E_4_) was also calculated using the calibration curves. Pristimerin and maytenin represented 2.53% (m/m) and 19.92% (m/m) in the sample, respectively. 

### 3.2. Cyclic voltammetric behavior of quinonemethide triterpenes and extract of Maytenus ilicifolia

**Figure 4:**
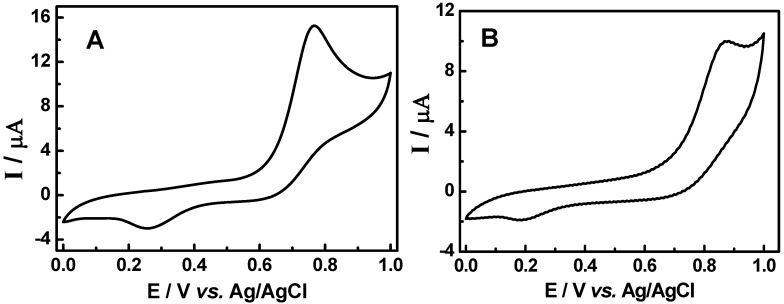
Cyclic voltammograms for oxidation of the 1.0 × 10^-3^ mol L^-1^ of (a) maytenin and (b) pristimerin in 0.1 mol L^-1^ of LiCl/methanol on the glassy carbon electrode. Scan rate: 50 mV s^-1^.

Typical cyclic voltammograms obtained for the two quinonemethide triterpenes; pristimerin and maytenin isolated from *Maytenus ilicifolia* are shown in Figure 4. The redox behavior of 1.0 × 10^-3^ mol L^-1^ pristimerin and maytenin in solution 0.1 mol L^-1^ LiCl in methanol at a glassy carbon electrode shows one defined peak at +0.78 V (Figure 4, Curve A) and +0.88 V (Figure 4, Curve B), respectively. This behavior is attributed to the oxidation of the phenolic groups to the quinone form [53]. For both quinonemethide compounds two cathodic peaks are observed on the reverse scan. The first one is very small and it is separated from the anodic peak by E_pa/2_-E_pc/2_ values of 53 and 60 mV, respectively, for maytenin and pristimerin, suggesting a process involving one electron [47]. But the relationship between I_pa_/I_pc_ presents values of around 0.29 (maytenin) and 0.19 (pristimerin), indicating that a chemical reaction is coupled to the electrochemical process, which is consuming the product of the anodic peak [54]. This new product is probably oxidized at a less positive potential around +0.25 V and +0.15 V for both compounds, respectively.

**Figure 5:**
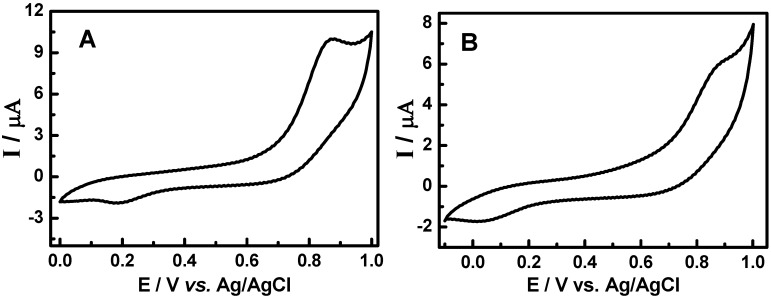
Cyclic voltammograms for oxidation of 1.0 × 10^-3^ mol L^-1^ of (A) rutin and (B) mixture of 5.0 × 10^-4^ mol L^-1^ pristimerin + 5.0 × 10^-4^ mol L^-1^ rutin in 0.1 mol L^-1^ of LiCl/methanol on the glassy carbon electrode. Scan rate: 50 mV s^-1^.

Taking into account that rutin can occur in leaves of the analyzed plant and presents known oxidative behavior and antioxidant properties [51,52,53], a cyclic voltammogram was recorded for rutin oxidation under the same experimental conditions defined previously for pristimerin and maytenin, as shown in Figure 5, Curve A. The oxidation of rutin in LiCl/methanol on a glassy carbon electrode presents a pair of redox peaks at potentials of +0.63 V/+0.40 V due to the oxidation of the catechol groups to the semiquinone form, after one electron transfer [51]. The E_pa/2_-E_pc/2_ values obtained from the cyclic voltammogram indicates that one electron is involved in the electrodic process [51] (n = 1.12). The ratio between peak currents are ip_c_/ip_a_ is close to 1, suggesting a classical reversible electrodic process behavior involving one electron and formation of a stable anion radical [51,53]. 

Rutin exhibits a lower oxidation potential then the quinonemethide triterpenes (pristimerin and maytenin) and also a reversible eletrodic process. This behavior confirmed the importance of the *ortho*-dihydroxy groups (ring B), carbonyl α,β-unsaturated, α,β-hydroxyketone groups in the ease of the oxidation. The form of the voltammograms is different for rutin and the triterpenes. The reversibility of the voltammetric curve can interfere with the antioxidant activity, since is indicative of radical stabilization after the initial oxidation steps [55].

It is known from the literature [56] that synergistic actions between synthetic, natural and synthetic, and natural antioxidants have been observed. This effect is defined as the combined action which results in an increased antioxidant potential greater than that expected for a simple additive effect. So, the occurence of quinonemethides and flavonoids in the sample of E_2_ and E_4_ extracts provides an excellent opportunity to use cyclic voltammetry to study their electrochemical behavior in *Maytenus ilicifolia* without previous separation of analytes.

In order to investigate the effect of high concentration of flavonol in the oxidative behavior of pristimerin, rutin was chosen as model. Rutin, (quercetin-3-*O-*rutinose), also known as vitamin P, is a type of flavonoid glycoside found in large amounts in plants which have attracted tremendous interest because of their free radical scavenging activities [57] and wide spectra of biological activities. 

Figure 5, Curve B shown the cyclic voltammogram obtained for a mixture of 2.0 × 10^-4^ mol L^-1^ of rutin and 5.0 × 10^-4^ mol L^-1^ of pristimerin in LiCl/methanol on glassy carbon electrode. The oxidation peak of rutin in the presence of pristimerin shifted 100 mV to less positive potential, indicating that is easier to oxidize under this experimental condition. In addition, a small shoulder is observed in the pristimerin peak height. Similar results have been observed in the literature, where the use of synergistic mixtures of antioxidants allow an increase in the antioxidant effectiveness with respect to the activity of the separate components [56].

**Figure 6:**
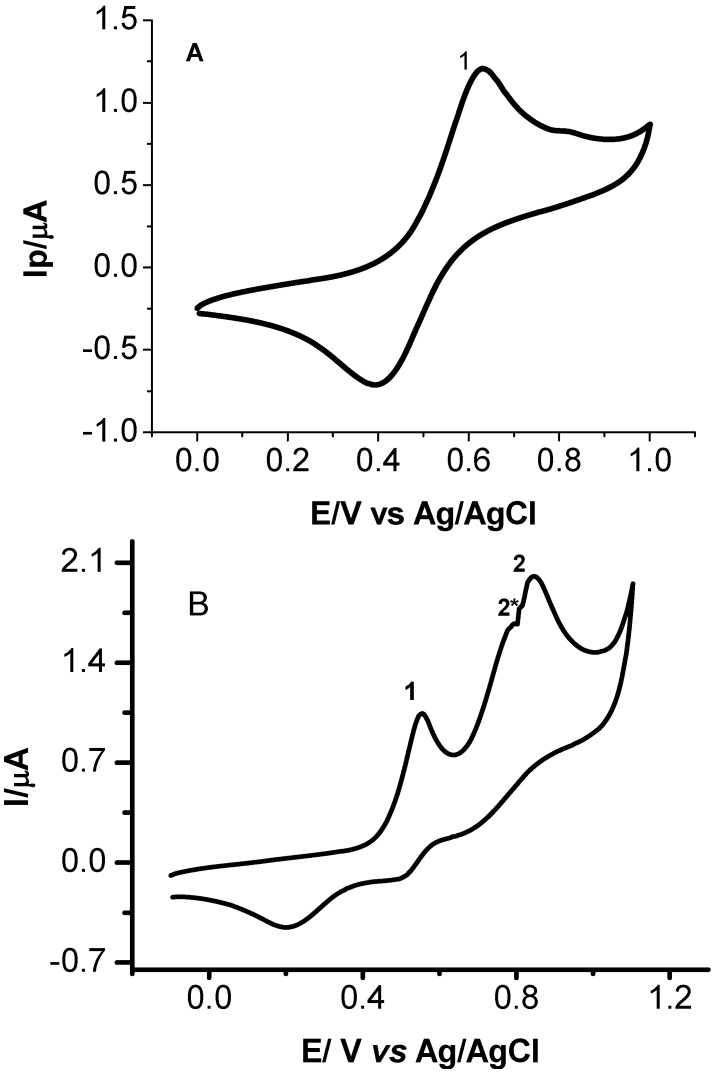
Cyclic voltammograms obtained for oxidation of sample containing 65 mg of (A) root bark the adult plant extract and (B)root seedlings extract in 0.1 mol L^-1^ of LiCl/methanol on the glassy carbon electrode. Scan rate: 50 mV s^-1^.

Typical cyclic voltammograms obained for oxidation of extracts containing 65 mg of (E_2_) root bark of adult plants (Curve A) and (E_4_) root seedlings (Curve B) in 0.1 mol L^-1^ LiCl/methanol on the glassy carbon electrode are shown in Figure 6. The cyclic voltammograms present the occurrence of a peak around +0.88 V, which could be attributed to the presence of pristimerin as preponderant metabolite in *Maytenus ilicifolia* extracts. Besides, there was no evidence of other flavonoids detectable due to their oxidative properties. 

**Figure 7:**
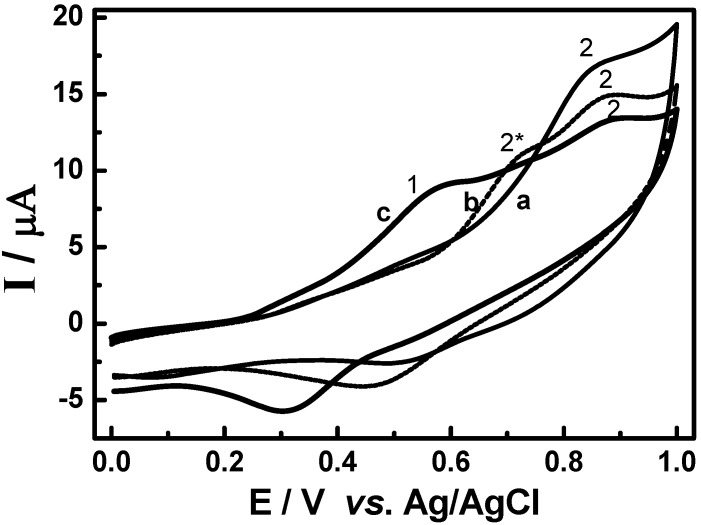
Cyclic voltammograms obtained for extract E_4_ from young plant before (a) and after addition of rutin at concentrations: b) 2.5 × 10^-4^ mol L^-1^; c) 5.0 × 10^-4^ mol L^-1^ in LiCl/methanol 0.1 mol L^-1^ on glassy carbon electrode. Scan rate = 50 mV s^-1^.

In order to confirm these results, studies of the effect of addition of rutin on the cyclic voltammetric oxidation of extracts seedlings of plant (E_4_) containing a preponderance of pristimerin (peak 1, Curve A) were also carried out. The results are shown in Figure 7. With increasing concentrations of rutin from 6.0 × 10^-5^ mol L^-1^ to 3.5 × 10^-4^ mol L^-1^ (Curve B), there is the decrease of the peak attributed to pristimerin (peak 1). Concomitantly the occurrence of a new extra peak at less positive potential (peak 2) is observed. This peak is shifted to a less positive potential when the rutin concentration is increased, as shown the peak 3 (Curves C) of Figure 7. This behavior suggests that there is a marked interaction between the flavonoids and pristimerin present in the extracts, which could result in an improved antioxidant activity, since the product is oxidized at less positive potential.

The voltammetric results obtained for isolated quinonemethide triterpenes (e.g., pristimerin and maytenin) and the rutin flavonoid have shown that the required oxidation potential decreases in the following sequence: rutin < maytenin< pristimerin.

These results were also compared with the DPPH assay [24] testing the antioxidant capacity. The stable free radical DPPH has an absorption maximum at 517 nm, which decreases upon reduction through reaction with an antioxidant compound. As expected, was observed to 100 μmol L^-1^ concentration the radical scavenging ability of 61.4 and 22.3% of the quinonemethide triterpenes maytenin and pristimerin, respectively. For the flavonoid rutin used as a model compound the value obtained is 82.0%, being around 3.7 times more potent than the quinonemethides, confirming the higher antioxidant capacity of the flavonoids. 

This antioxidant activity observed in the quinonemethide triterpenes is characteristic of phenol/dienonic system and the double conjugated bond quinonemethides system present on the aromatics rings A and B of the chemical structure. The free radical scavenging activity of quinonemethide triterpenes is mostly due to the α,β-unsaturated carbonyl moiety with extended conjugation through ring B. The free radical scavenging ability the model compound, rutin, is due to catechol moiety present in the ring B of rutin the C-2 and C-3 double bond [58]. Thus, the DPPH test confirms the electrochemical oxidation behavior.

### 3.3. Evaluation of the extracts E_2_ and E_4_ via HPLC with electrochemical detection

**Figure 8:**
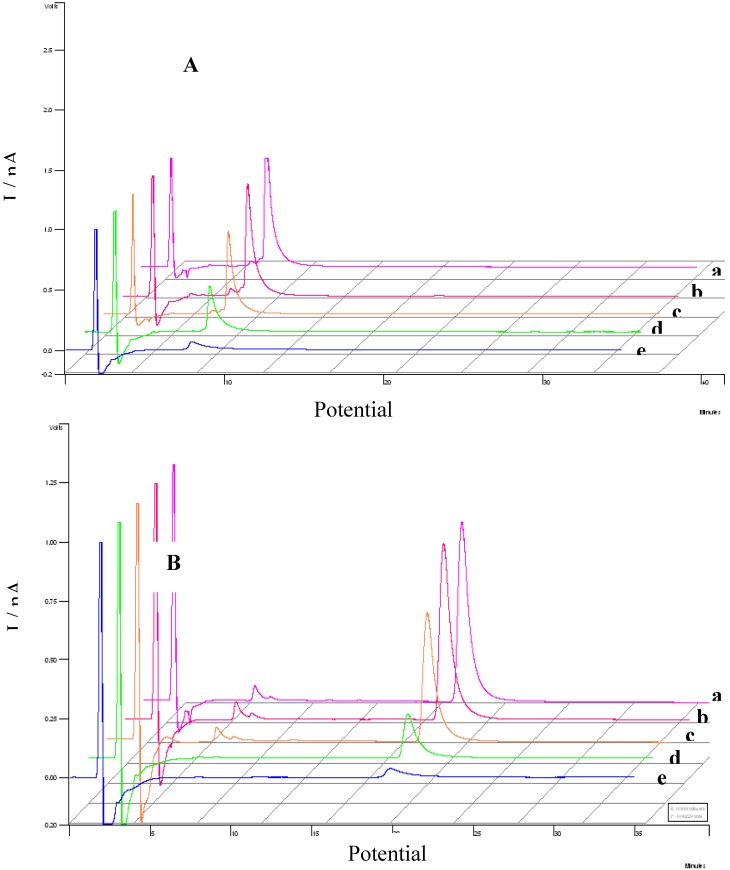
Effect of applied potential E_ox_ on chromatograms recorded for standard sample of maytenin (A) and pristimerin (B). Separation carried out on a Phenomenex Gemini C_18_ analytical column, (85:15) MeCN-H_2_O (HOAc aqueous 0.3%), mobile phase, 1.0 mL min^‑1^. ED conditions: digital filter set at 0.1 s; 30 °C; 0% offset; range 5ηA; E_ox_: a) 0.8 V; b) 0.7 V; c) 0.6 V; d) 0.5 V; e) 0.4 V.

On the basis of the results indicating that the electrochemical response can be correlated with antioxidant activities, the combination of both HPLC and the response at a certain oxidation potential were investigated by using HPLC coupled to electrochemical detection (HPLC-ED). In order to evaluate the antioxidant activity of the extracts from root barks of adult plant (E_2_) and root of seedlings (E_4_) extracts electrochemical studies were carried out by testing the samples and comparison with standards. Samples of each extract and standards were submitted to chromatographic separation at an applied potential of +0.8 V testing stationary phase, the mobile phase and the flux speed. The chromatograms obtained for standards maytenin and pristimerin isolated from *Maytenus ilicifolia* are shown in Figure 8 (Curves A and B), respectively. 

**Figure 9:**
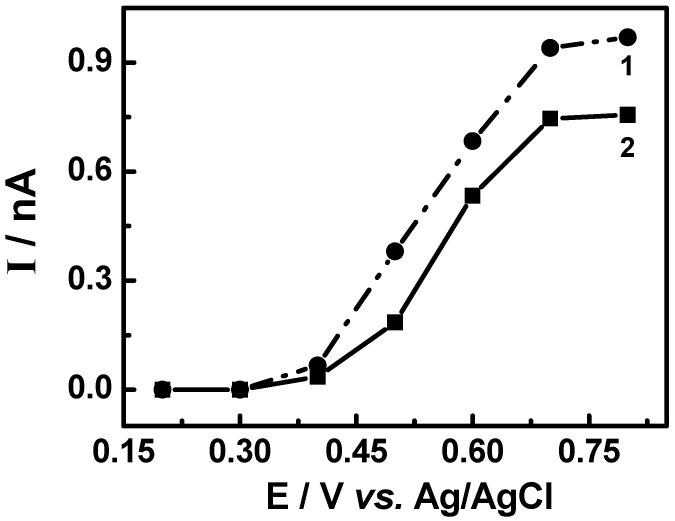
Hydrodynamic voltamograms obtained during oxidation of maytenin (**1**) and pristimerin (**2**) isolated from *M. ilicifolia*. Separation carried out on a Phenomenex Gemini C_18_ analytical column, (85:15) MeCN-H_2_O (HOAc aqueous 0.3%), mobile phase, 1.0 mL min^-1^. ElCD conditions: digital filter set at 0.1 s; 30 °C; 0% offset; range 5 ηA; E_ox_: a) 0.8 V; b) 0.7 V; c) 0.6 V; d) 0.5 V; e) 0.4 V.

Figure 9 illustrates the hydrodynamic voltamograms obtained from current values of peaks identified in the chromatogram to the standards of maytenin and pristimerin vs applied potential changing from +0.2 to +0.8 V. For both triterpene standards it was observed that the identification of any of the compounds occurs only at potential greater than +0.5 V. However, the maximum current is obtained in potential greater than or equal to +0.7 V. The selection of the applied potential is an important parameter, since it directly affects the intensity of the response in the chromatogram. Thus, a value of E_app_ = +0.8 V was chosen as the optimum condition to determine each compounds in the extracts. 

**Figure 10:**
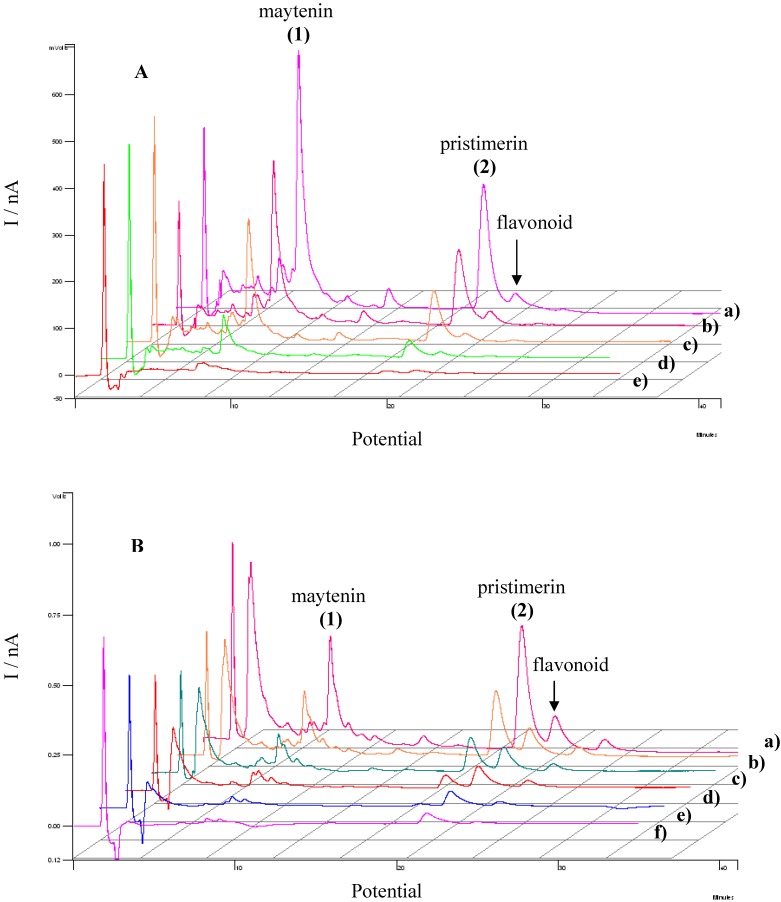
Effect of potential on HPLC-ED chromatograms obtained from (A) root barks the adults plants (E_2_) and (B) root of seedlings (E_4_) of *Maytenus ilicifolia* extract. Separation carried out on a Phenomenex Gemini C_18_ analytical column, (85:15) MeCN-H_2_O (HOAc aqueous 0.3%), mobile phase, 1.0 mL min^-1^; digital filter set at 0.1 s; 30 °C; 0% offset; range 5ηA; E_ox_: a) 0.8 V; b) 0.7 V; c) 0.6 V; d) 0.5 V; e) 0.4 V.

The relative responses for each triterpene component (maytenin and pristimerin) and also the flavonol contents detected in both extracts from root barks of adult plant (E_2_) and root of seedlings (E_4_) measured at different potentials is shown in Figure 10 (curves A and B), respectively.

The chromatograms obtained for extract obtained from root barks of adult plant (E_2_) presented two defined peaks with high intensity at retention times of 5.30 and 16.42 min, attributed to the components maytenin and pristimerin. Both peaks are also seen in the chromatograms obtained for extract from root of seedlings (E_4_) extract at retention times of 4.41 and 16.21 min, too. In addition, both samples exhibits extra peaks around a retention time of 18.51 min for extract from root barks of adult plant (E_2_) and 18.23 min for root of seedlings (E_4_) extract attributed to the flavonols and the occurrence of a new unidentified peak at a retention time of 22.9 min in root of seedlings (E_4_) extract, but with potential antioxidant activity, too. 

The analysis of the potential under the chromatogram obtained from the extracts obtained indicates that the potential necessary to detect both triterpenes occurs at a smaller oxidation potential, +0.5 V in the extract than for individual compounds. The hydrodynamic voltammogram obtained for extracts from root of seedlings (E_4_) also presented a shift of around 100 mV to less positive values in the oxidation potential used to detect pristimerin. These results indicates that extracts from root of seedlings (E_4_) flavonols can be oxidized at lower potentials, such as +0.4 V and +0.3 V for maytenin and pristimirin, when compared to triterpenes in the isolated sample. This behavior reveals that in the extracts E_4_ the antioxidant activity is potentiated, which could be attributed to a synergistic interaction between the antioxidant substances present in the extract from root of seedlings (E_4_) and other components that promotes the oxidation of flavonols at a lower potential. The same effect is corroborated with the synergism observed previously in the voltammetric studies, using rutin as model compound.

## 3. Experimental

### 3.1. Instrumental

*HPLC-UV-DAD analyses*: HPLC analyses were performed on a Varian Pro-STAR pump model 240, with a diode array ultraviolet detector (HPLC-UV-DAD). Separation was achieved on a Phenomenex Gemini C_18_ 5µ reverse phase column (25 cm × 4.6 mm) in isocratic mode using MeCN:H_2_O (85:15, with 0.5% HOAc) at a flow of 1.0 mL min^-1^ as mobile phase. The characteristic retention times for compounds **1** and **2** under these HPLC conditions were 7.3 and 17.8 min. The UV absorbance was set at 254 and 420 nm.

*Preparative HPLC*: Separations were performed on a Varian PROSTAR, using a Phenomenex-Luna 10 µm (21.2 × 250 mm) reversed phase column, eluted with MeOH:H_2_O (9:1,with 0.1% HOAc) and detection set at 420 nm.

*HPLC-EICD*: The analytical methodology was developed using a VARIAN STAR 9090 pump coupled to an EICD eletrochemical detector (Rotterdam, The Netherlands) composed of a glassy carbon working electrode, an Ag/AgCl reference electrode and a Pt electrode. The optimal potential to evaluate the pristimerin and maytenin standards was obtained from a hydrodynamic voltammogram recorded from evaluation of peak areas *vs* applied potential *vs* Ag/AgCl. Separation of the analytes was carried on a Phenomenex Gemini C_18_ column (5 µm, 25 cm × 4,6 mm), using isocratic mode MeCN/H_2_O (85:15) containing acetic acid (0.3% v/v) as mobile phase. Each standard compound (20 µL) was injected at several working electrode potentials ranging from +0.4 to +0.8 V. 

*Electrochemical assay*: Electrochemical measurements were carried out using a Potentiostat/Galvonostat Autolab PGSTAT 30. A three-electrode system with a working electrode of glassy carbon, an Ag/AgCl reference electrode and a platinum wire as auxiliary electrode were used. The working electrode (3 mm diameter) was polished with alumina (0.3 μm, BUEHLER), and was then washed with water, and dried at room temperature before use. Methanol was used as solvent and 0.1 mol L^-1^ of LiCl (Merck) as the supporting electrolyte. The flavonoid rutin (Aldrich) was used as reference compound due its well known oxidation properties [47,48]. The solutions of compounds were prepared daily to concentration of the 1.0 × 10^-3^ mol L^-1^ in LiCl/methanol. The solutions were then deoxygenated with nitrogen for 10 min and the cyclic voltammograms were carried out at scan rate of 50 mV s^-1^.

### 3.2. Plant material

The root barks from adults and three years old seedlings (voucher number HPMU-0266) were kindly offered by Drª. Ana Maria Soares Pereira (UNAERP, Ribeirão Preto, SP, Brazil), and were taken from specimens originating from seedlings of *Maytenus ilicifolia* raised in UNAERP̓s greenhouse. 

### 3.3. Extraction and isolation

The root barks of seedlings of *Maytenus ilicifolia* (E_4_) were frozen in liquid N_2_. A sample of 12.90 g of the plant material was extracted using 100 mL of dichloromethane in a sonicator. The resulting extract was filtered and after evaporation produced 0.060 g of crude extract. 

Root barks of adult plants of *Maytenus ilicifolia* (E_2_) were utilized for isolation of the quinonemethides maytenin and pristimerin. This material was dried in an oven at 40 °C and pulverized in a mill, to give 191.00 g of the dried material. The powder was submitted to extraction with dichloromethane with the aid of a sonicator bath. Afterwards, the exctract was filtered and evaporated, resulting in a mass of 2.60 g. Sample of 0.200 g of the exctract, was fractionated by Prep. TLC, eluted with hexane- EtOAc (8:2), obtaining five fractions. Fractions F_5_ (0.072 g) and F_3_ (0.040 g), were submitted to preparative HPLC eluted with MeOH-H_2_O (9:1 + 0.1% HOAc ) at wavelength of 420 nm and flow rate of 10 mL min^-1^ to yield pristimerin (0.055 g) and maytenin (0.030 g). The compounds were identified by comparison of their ^1^H-NMR, ^13^C-NMR and ES-MS data with literature values [13]. The isolated and characterized compounds were used as standards.

The samples and standards were submitted to purification using a Sep-Pak^®^ (ODS) cartridge using 100% methanol, after which the solution were analyzed directly by HPLC-UV-DAD and HPLC-EICD. 

## 4. Conclusions

The analysis by HPLC-DAD of the *Maytenus ilicifolia* extracts obtained from root barks of adult plants (E_2_) and the roots of the seedlings (E_4_) indicates the presence of quinonemethide triterpenes and/or phenolic compounds as the main constituent metabolites. The quantitative analysis of the extracts E_2_ and E_4_ showed that the quinonemethide triterpene pristimerin is the major component of both extracts.

The quinonemethide triterpenes **1** and **2**, as well as the flavonoids accumulated in the root barks of *Maytenus ilicifolia,* were evaluated by HPLC-ED and cyclic voltammetry. The results indicate the co-occurrence of two classes of compounds could influence the antioxidant potential of the respective extract. The proposed methodology using two different techniques (HPLC-ED and CV) helps the understanding of the antioxidant properties and could be an alternative tool to evaluate the possible synergistic effects of flavonoids and quinonemethides in *Maytenus ilicifolia* based on the oxidation potential required for their electrochemical oxidation processes. Both methods are simple and efficient for detecting and to quantifying phenolic antioxidants in complex matrixes.
